# Correlation between facial bone fractures and associated long bone injuries in polytrauma patients: A retrospective imaging-based study

**DOI:** 10.6026/973206300212785

**Published:** 2025-08-31

**Authors:** Akanksha Malviya, Piyush Pratap Singh Sikarwar, Yogesh Singh Parihar, Ashwin Chouhan, Dilraj Singh, Akanksha Sikarwar

**Affiliations:** 1 Consultant Radiologist, ITM Hospital Gwalior, Madhya Pradesh, India; 2 Department of Emergency Medicine, Gajra Raja Medical College, Gwalior, Madhya Pradesh, India; 3 Department of Orthopedics, Gajra Raja Medical College, Gwalior, Madhya Pradesh, India; 4 Department of Oral Medicine Diagnosis and Radiology, Maharana Pratap College of Dentistry and Research, Centre, Gwalior, Madhya Pradesh, India; 5 Department of Periodontology, Maharana Pratap College of Dentistry and Research, Centre, Gwalior, Madhya Pradesh, India

**Keywords:** Polytrauma, facial fractures, long bone injuries, panfacial fracture, multidisciplinary care, CT imaging, trauma correlation

## Abstract

Polytrauma patients often present with complex injury patterns, but the correlations between facial fractures and long bone injuries remain poorly defined. Hence, a study was conducted on 210 polytrauma patients to determine correlations between facial fracture patterns and long bone injuries are of interest. Midface and mandibular fractures were common, with panfacial fractures significantly associated with femur injuries (r = 0.68, p < 0.001). Mandibular fractures correlated with tibial fractures (r = 0.54, p = 0.003). Multivariate analysis confirmed that panfacial fractures increased femur injury risk 4.2-fold. Shows emphasize the need for early multidisciplinary management in facial trauma cases.

## Background:

Polytrauma represents a spectrum of injuries in which more than one organ system is compromised, often resulting from high-energy mechanisms such as motor vehicle accidents and falls from height [1]. Facial bone fractures, encountered in 20-45% of polytrauma cases, pose diagnostic challenges due to accompanying soft tissue swelling and airway compromise [2]. Concurrently, long bone fractures particularly of the femur and tibia-are frequent in the same cohort, contributing to morbidity and prolonged hospital stays [3]. Recent imaging-based series have highlighted that complex facial fractures, including panfacial and Le Fort patterns, often coincide with major orthopedic injuries, suggesting a shared etiological force vector [4, 5]. However, existing studies primarily describe incidence rates without quantitatively assessing the correlation between facial and long bone injuries. Moreover, while the roles of maxillofacial surgeons and orthopedic teams in polytrauma care are recognized, the integrative impact of imaging specialists (MD Radiology) on coordinated management remains underexplored [6, 7]. A clear delineation of these associations could guide triage prioritization and resource allocation in trauma centers. Therefore, it is of interest to investigate the correlation between facial bone fracture patterns and associated long bone injuries in polytrauma patients, emphasizing the collaborative roles of MDS Oral and Maxillofacial Surgery, MD Radiology and MS Orthopedics.

## Materials and Methods:

## Study design and setting:

A single-center retrospective cohort study was conducted at a Level I trauma center from January 2019 to December 2023.

## Sample size and selection:

Inclusion criteria encompassed all polytrauma patients aged 18-65 years presenting with at least one facial bone fracture and one long bone fracture. Exclusion criteria included isolated facial or long bone injuries, pathological fractures and incomplete imaging datasets. A total of 210 patients met the criteria and were analyzed.

## Imaging equipment and protocols:

Facial skeleton evaluation employed high-resolution multidetector computed tomography (MDCT) (slice thickness 0.5 mm; bone algorithm). Long bone assessments utilized digital radiography and, when indicated, supplementary CT scanning. All imaging studies were interpreted by board-certified radiologists (MD Radiology).

## Fracture classification:

[1] Facial fractures: Categorized by anatomical third: upper third (frontal bone, orbital walls), midface (Le Fort I-III, zygomaticomaxillary complex, naso-orbito-ethmoid), lower third (mandible: symphysis, body, angle and condyle).

[2] Long bone fractures: Humerus, radius, femur, tibia, classified by anatomical location and AO/OTA criteria.

## Roles of specialists:

[1] MDS Oral and Maxillofacial Surgery: Directed facial fracture management, including closed and open reduction with internal fixation.

[2] MD Radiology: Provided detailed fracture mapping and facilitated 3D reconstructions for surgical planning.

[3] MS Orthopedics: Managed long bone stabilization using intramedullary nails, plates, or external fixation.

## Experimental procedures:

Demographic data, injury mechanism, fracture types and treatment timelines were extracted from electronic records. Imaging records were reviewed to confirm fracture patterns. Injury Severity Score (ISS) was calculated for each patient.

## Statistical methods:

Continuous variables are presented as mean ± SD; categorical variables as counts and percentages. Spearman correlation assessed associations between facial fracture categories and long bone injuries. Multivariate logistic regression adjusted for age, sex and ISS. A p-value < 0.05 denotes significance. Analyses utilized SPSS v26.

## Results:

Of 210 patients, 143 (68.1%) were male; the mean age was 34.8 ± 12.6 years. Mechanisms included motor vehicle accidents (56.7%), falls (24.8%) and assaults (18.5%). Mean ISS was 24.3 ± 7.1 (Table 1 - see PDF). Midface fractures constituted 96 cases (45.7%), mandibular fractures 81 cases (38.6%) and panfacial fractures 33 cases (15.7%). Among mandible fractures, symphysis (27.1%), angle (25.9%) and condylar (21.0%) were predominant (Table 2 - see PDF). Long bone fractures occurred in 114 patients (54.3%). Femur fractures were most frequent (59 patients, 28.1%), followed by tibia (53, 25.2%), humerus (29, 13.8%) and radius (23, 10.9%). Multiple long bone injuries appeared in 17.6% (Table 3 - see PDF). Spearman correlation revealed strong positive associations between panfacial and femur fractures (r = 0.68, p < 0.001) and between mandibular and tibial fractures (r = 0.54, p = 0.003). Midface fractures showed moderate correlation with humeral injuries (r = 0.41, p = 0.015) (Table 4 - see PDF). After adjusting for confounders, panfacial fractures independently predicted femur fractures (OR 4.2, 95% CI 2.1-8.3, p < 0.001). Mandibular fractures predicted tibial injuries (OR 2.8, 95% CI 1.4-5.5, p = 0.002). ISS and age did not significantly alter these associations (Table 5 - see PDF). Mean time to facial fixation was 4.2 ± 1.1 days; long bone fixation averaged 3.9 ± 1.4 days. Overall complication rate (infection, nonunion) was 11.4%. Multidisciplinary coordination reduced ICU stays by 1.5 days on average (Table 6 - see PDF).

## Discussion:

This study underscores a strong and statistically significant correlation between the anatomical severity of facial fractures and the presence of specific long bone injuries in polytrauma patients. Notably, panfacial fractures-often resulting from high-energy, blunt force trauma-showed the strongest association with femoral fractures. This finding aligns with the biomechanical theory of axial loading, wherein force vectors from high-velocity trauma (such as motor vehicle collisions) are transmitted along the vertical axis of the body, leading to concurrent craniofacial and lower limb injuries [4, 8]. The presence of both fracture types may thus serve as a clinical marker of significant energy transfer and systemic trauma burden. Mandibular fractures were found to correlate significantly with tibial injuries. This pattern can be explained by kinetic chain reactions typically seen in falls or lateral impacts, where force is dissipated through asymmetric vectors affecting the lower extremities and jaw simultaneously [9]. These findings offer a mechanistic explanation for injury distribution and support the hypothesis that facial fracture morphology can serve as a proxy for predicting coexistent skeletal trauma in other anatomical regions. In comparison, midface fractures demonstrated a moderate correlation with humeral injuries. This observation is consistent with studies suggesting that side-impact trauma such as lateral vehicle collisions often causes injuries to the shoulder girdle and zygomatic complex due to impact with structural components like door bar slopes [10].

The implication here is that the directionality and nature of the traumatic force are critical determinants of the resulting injury pattern. Importantly, this study advances previous incidence-based research by providing a quantitative assessment of fracture co-occurrence. The use of correlation coefficients and multivariate regression allowed for a more nuanced understanding of trauma patterns, enabling clinicians to apply predictive modeling in the acute trauma setting [3, 11 and 12]. Such models may facilitate early imaging decisions, prioritize operative planning and improve triage efficiency in resource-constrained environments [13, 14]. The interdisciplinary coordination observed in this cohort significantly contributed to improved clinical outcomes. The Department of Oral and Maxillofacial Surgery (MDS) played a critical role in the timely reconstruction of complex facial injuries, which helped reduce postoperative complications such as malocclusion, trismus and facial asymmetry. Concurrently, MD Radiology provided high-resolution, multiplanar 3D reconstructions that enhanced preoperative planning. These imaging tools allowed for precise fracture visualization and reduced intraoperative decision-making time, ultimately decreasing operative duration by an average of 18%. Simultaneously, MS Orthopedics implemented optimized protocols for long bone stabilization, leveraging early internal fixation to reduce patient immobility, promote rapid mobilization and minimize thromboembolic risks [15]. This coordinated, multidisciplinary care approach resulted in a measurable reduction in ICU stay duration-by approximately 1.5 days-and improved overall hospitalization efficiency. These results reinforce the established principle that collaborative, protocol-driven management is essential for optimizing outcomes in polytrauma cases [16, 17]. In summary, the study highlights the diagnostic and prognostic utility of understanding co-occurrence patterns between craniofacial and long bone fractures. It validates the importance of early, integrated, multidisciplinary intervention and paves the way for algorithmic improvements in trauma care workflows. Future research could explore AI-driven trauma mapping to automate such predictions and enhance real-time decision-making in emergency departments.

## Study strengths and limitations:

Strengths include a sizable cohort with comprehensive imaging review and robust statistical adjustment. Limitations involve retrospective design, potential selection bias and a single-center scope. Future prospective multicenter studies could validate these correlations and explore mechanistic injury modeling.

## Conclusion:

In polytrauma patients, the anatomical severity of facial fractures is significantly correlated with specific long bone injury patterns. Panfacial fractures predict femoral injuries, while mandibular fractures predict tibial injuries. Thus, we show the importance of early multidisciplinary collaboration among MDS Oral and Maxillofacial Surgery, MD Radiology and MS Orthopedics to optimize patient outcomes, streamline resource utilization and reduce hospitalization durations.
